# Barriers and enablers of research participation among undergraduate health students in Sub-Saharan Africa: a systematic review

**DOI:** 10.1186/s12909-026-08681-2

**Published:** 2026-02-17

**Authors:** Archibong Edem Bassey, Prosper Ayenmo Kanu, Uchenna Frank Imo, Usoro Udousoro Akpan, Yusuff Adebayo Adebisi, Chinaza Duke Nwosu

**Affiliations:** 1 Warwick Medical School, University of Warwick, Coventry, CV47AL UK; 2 Department of Public Health, University of Calabar, Calabar, Nigeria; 3 College of Social Sciences, University of Glasgow, Glasgow, UK; 4 Alliance Manchester Business School, University of Manchester, Manchester, UK

**Keywords:** Undergraduate health students, Research participation, Barriers, Enablers, Sub-Saharan Africa, Research capacity building.

## Abstract

**Background:**

Research participation among undergraduate healthcare students (UHS) is widely recognized as key to their academic and professional development, as well as to developing future competent health workforce. However, in Sub-Saharan Africa (SSA), the participation of undergraduate health students in research remains limited. This review aims to systematically explore the barriers and enablers to research participation among UHS in SSA.

**Methods:**

A systematic search was conducted across MEDLINE, Embase, Scopus, CINAHL, and Web of Science databases for studies published in English from 2000 to 2024. Studies were screened and assessed for quality using the Mixed Methods Appraisal Tool. Data were synthesized through narrative synthesis.

**Results:**

Ten studies from Uganda, South Africa, Namibia, Rwanda, Sudan, and Nigeria were included, with quality ratings from moderate (60%) to very high (100%). Reported research activities covered early (conceptualisation, ethics approval), middle (data collection, analysis), and late (dissemination, publication) phases. Barriers were clustered into: (1) resource constraints; (2) time and curriculum pressures; (3) inadequate mentorship; (4) knowledge and skills gaps; (5) administrative and ethical hurdles; (6) perceptions, attitudes to research and (7) Gender, Language and Cultural barriers. Enablers included: (1) early research exposure; (2) strong mentorship; (3) access to funding/resources; (4) research skills training; (5) individual motivation; and (6) institutional/peer support. Some factors such as supervision and resources were reported as both barriers and enablers.

**Conclusion:**

UHS in SSA face intertwined structural, institutional, and personal challenges to research participation. Strengthening mentorship, integrating research early into curricula, improving access to resources, and providing skills training may enhance engagement and build sustainable research capacity in the region.

**Trial registration:**

This review was prospectively registered on PROSPERO database (PROSPERO 2024 CRD42024581644).

## Introduction

Research has been a veritable tool to increasing the understanding of diseases, disease mechanisms and offering evidence-based strategies to protect public health [1]. With the presence of epidemics and pandemics in recent years, such as HIV, COVID-19 and M-pox [2, 3], there is an increased need for research to bridge knowledge gap and provide appropriate tools to tackle these rising public health challenges [4]. Sub-Saharan Africa (SSA), with a population of approximately 1.26 billion, carries a disproportionately high share of the global disease and mortality burden, accounting for 25% of the world’s total [5]. This situation is largely driven by limitations in the quality of healthcare services across the region, further compounded by a critical shortage of locally generated research evidence to inform effective healthcare interventions and policies [6]. Africa as a continent has just 198 researchers per million people, compared to over 4,000 per million in countries like the United Kingdom and the United States [7, 8]. Moreover, no African nation has met the African Union’s target of allocating 1% of its gross domestic product to research and development [7]. This gap has led many African nations to rely heavily on findings from high-income countries, whose disease profiles and levels of medical advancement differ significantly from those in SSA [6]. Such reliance can lead to poor health outcomes, as seen during the Ebola outbreak in West Africa, where a lack of skilled local clinical researchers contributed to increased disease transmission and mortality rather than effective containment [6, 9].

Consequently, there is a growing emphasis across SSA on strengthening undergraduate engagement in research activities [5]. Undergraduate Health Students (UHS) are widely regarded as the main pipeline of future healthcare professionals, including clinicians and health research scientists [6]. The undergraduate period presents a strategic window for developing research competencies, fostering scientific curiosity, and nurturing positive attitudes and behaviours. These can promote sustained involvement in research throughout their careers [10]. There are several examples of students who were engaged in research as undergraduates, who have gone on to make great achievements [10].

Although several African countries have implemented measures such as integrating research methodology early into academic curricula to strengthen research capacity [6], for most undergraduate health related programs, students often only get to experience research as components of the traditional final/penultimate year thesis [9]. UHS research participation can go beyond this norm and can be integrated across the curriculum as early as their first year [11, 12]. Earlier and diverse exposure to research can result in the development of cognitive skills including critical thinking and reasoning, which has been linked to higher self-efficacy, and better outcomes for patients and health system [10]. It can also foster increased competencies in academic writing, scientific communication, data collection and analysis, which can increase their likelihood of pursuing research careers, contributing to publications, and implementing evidence-based practices in clinical (and a variety of) settings [6, 9]. Furthermore, in the context of Sub-Saharan Africa, where strengthening indigenous research capacity represents a strategic imperative for addressing regional health challenges, nurturing research engagement at the undergraduate level establishes essential foundations for sustainable research ecosystems [13, 14].

As highlighted, the benefits of research participation are vast, and in other climes, involving undergraduate students in research has been viewed as a high impact practice [15, 16]. However, the current understanding of the barriers and enablers of research participation among undergraduate health students in SSA remains fragmented, with studies typically focusing on specific health disciplines, single institutions or particular countries within the vast and diverse SSA region [17]. For instance, Awofeso et al. [18] conducted a similar study but focused only on medical students at the College of Medicine, University of Lagos. In contrast, another study [6] focused on 12 universities within Uganda offering health professional courses. Beyond understanding barriers and enablers, it is equally important to understand how UHS participate in research and thus examine the types and forms of research activities they undertake. To our knowledge, there has been no comprehensive synthesis of evidence as regards undergraduate health student’s perspectives on research participation in SSA. This knowledge gap may limit the development of evidence-based strategies to build research capacity, starting at the undergraduate level.

### Aim/objectives

To address this evidence gap, our systematic review aimed to synthesize evidence on the barriers and enablers to research participation among undergraduate health students across SSA. In doing this, we also examined the various forms and/or types of research activities undertaken by undergraduate health students in Sub-Saharan Africa.

The findings provide recommendations for enhanced undergraduate health students’ participation in research. These recommendations also seek to strengthen research capacity building at the undergraduate stage in the training of health professionals in SSA.

## Methods

We conducted this review following the Preferred Reporting Items for Systematic Reviews and Meta-Analysis (PRISMA) guidelines [19]. This review protocol was registered on PROSPERO (ID: CRD42024581644) on 23rd August 2024.

### Eligibility criteria

#### Inclusion criteria

Studies were included if they involved undergraduate students enrolled in health-related programs (medicine, nursing, pharmacy, public health, and allied health professions) in Sub-Saharan African countries and collected primary data using qualitative, quantitative, or mixed-methods approaches to explore barriers and enablers affecting research participation among this population.

#### Exclusion criteria

Studies were excluded if they featured only in-service health professionals, postgraduate students or undergraduate students not enrolled in a health-related discipline. We also excluded studies not conducted in Sub Saharan Africa, and other studies such as reviews, opinion pieces, commentaries, editorials, and books.

### Search strategy

A systematic search was conducted across five databases, MEDLINE, Embase, Scopus, CINAHL, and Web of Science databases for studies published in English from 2000 to 2024. The initial search was conducted in August 2024, and preliminary findings were presented at a conference in November 2024. This search was updated in December 2024 to cover the full year, 2024. The decision to limit the start of the search to articles from the year 2000 onwards was informed by the presence of key transformative commitments in education in that year, particularly those made at the World Education Forum held in Dakar, Senegal in April 2000 [20]. This forum, attended by world leaders, led to the birth of the Dakar Framework for Action. This framework is aimed at driving the achievement of education for all, particularly in Sub Saharan Africa and other contexts. Further, priority areas of focus in the framework mapped out for sub-Saharan Africa include improving quality and access to education, improving the quality and relevance of education, institutional and professional capacity building, and improving partnership. These areas demonstrate relevance to the aim of this systematic review.

The search included 3 main construct blocks using the PCC framework, combined with Boolean operators: (1) Population terms capturing undergraduate students in health-related disciplines, (2) Concept terms addressing both barriers and enablers to participation in research, and (3) Context terms capturing Sub-Saharan Africa and all individual country names within the region. See example of a search string in Table 1 below.

**Table 1:** Search string carried out on SCOPUS database

S/*N*	Search Terms
1	TITLE-ABS-KEY (undergraduate* OR “medical student*” OR “nursing student*” OR “health science* student*” OR “healthcare student*” OR “health profession* student*” OR “public health student*” OR “pharmacy student*” OR “allied health* student*” OR “university student*” )
2	TITLE-ABS-KEY ( ( barrier* OR obstacle* OR challeng* OR imped* OR hinder* ) OR ( enabl* OR foster* OR facilitat* OR motivat* OR promot* ) )
3	TITLE-ABS-KEY ( research )
4	TITLE-ABS-KEY ( participat* OR involv* OR engag* OR le? d* OR partner* or collaborat* )
5	TITLE-ABS-KEY ( “Sub-Saharan Africa” OR “Sub Saharan Africa” OR SSA OR Angola OR Benin OR botswana OR “Burkina Faso” OR burundi OR cameroon OR “Cape Verde” OR “Central African Republic” OR chad OR comoros OR “Congo” OR “Democratic Republic of Congo” OR drc OR “Republic of Congo” OR “Cote d&apos;Ivoire” OR “Ivory Coast” OR djibouti OR “Equatorial Guinea” OR eritrea OR ethiopia OR gabon OR gambia OR ghana OR guinea OR “Guinea-Bissau” OR kenya OR lesotho OR liberia OR madagascar OR malawi OR mali OR mauritania OR mauritius OR mozambique OR namibia OR niger OR nigeria OR rwanda OR “Sao Tome and Principe” OR senegal OR seychelles OR “Sierra Leone” OR somalia OR “South Africa” OR “South Sudan” OR sudan OR swaziland OR eswatini OR tanzania OR togo OR uganda OR zambia OR zimbabwe )
6	#1 AND #2 AND #3 AND #4 AND #5 AND PUBYEAR > 2000 AND PUBYEAR < 2025 AND ( LIMIT-TO ( LANGUAGE, “English” ) )

### Study screening and selection

All identified records were collated and imported into Covidence systematic review software, where duplicate records were identified and removed. Following that, three reviewers (AEB, UUA and CDN) independently screened the titles and abstracts of the deduplicated records against the eligibility criteria. Afterwards, the full texts of studies deemed potentially eligible during the initial screening were retrieved and independently assessed by the same three reviewers. At each stage, any discrepancies between reviewers were resolved through discussion and consensus, with a fourth reviewer (AYA) serving as an umpire when necessary. Through this process, the number of studies excluded at each stage was documented in a PRISMA flow diagram.

### Data extraction

After finalizing the list of included studies, a data extraction form was developed and utilized to systematically collect information from each study. Data extracted included study characteristics (authors, publication year, country, study design, sample size), participant demographics (age range, gender distribution, academic year, health discipline), and specific details about research activities, barriers, and enablers to research participation among undergraduate health students in Sub-Saharan Africa.

### Quality appraisal

Studies were appraised for quality using the Mixed Methods Appraisal Tool (MMAT) Version 2018 [21]. The MMAT was selected for its versatility in evaluating various study designs including qualitative, quantitative and mixed methods.

We meticulously assessed each study against key methodological criteria specific to its design type as outlined in the MMAT framework. Each criterion was scored using a three-point scale (‘yes’, ‘no’, or ‘cannot tell’), with individual study quality scores calculated as the percentage of criteria receiving a “yes” response. We subsequently categorized the overall quality of studies using a six-tier classification system: 0% (no quality), 20% (very low quality), 40% (poor quality), 60% (moderate quality), 80% (good quality), and 100% (very high quality). Any discrepancies or disagreements in quality assessments were resolved through reviewer discussions. Following recommendations of reporting results of the MMAT, results were presented within our study characteristics table, in a column that shows the overall score for each study, providing transparent evaluation of the methodological rigor underpinning our synthesis. In reporting the quality appraisal scores of included studies, no study was excluded based on the scores. This was to ensure that data were collected from all studies related to the research question.

### Data synthesis

We employed a narrative synthesis approach, guided by the framework proposed by [22], adapted specifically for our review objectives. This approach to synthesis focused on integrating findings from the various study designs to provide a comprehensive understanding of the research topic.

This began with a preliminary synthesis, where AEB, PAK, and UFI independently created textual descriptions of the included studies, and developed tables to comprehensively summarize study characteristics, participant demographics and key research findings. These reviewers then used systematic vote counting to identify frequently reported barriers and enablers to research participation across the studies. This preliminary stage also allowed for the extraction of data relevant to other objectives- particularly the various ways UHS participate in research, and the types of research activities they engage in. The research activities were grouped into phases following our adaptation of the Whittemore and Melkus’s framework [23] on the research process.

The same three reviewers then explored relationships in the data through collaborative thematic analysis. Here, similar barriers and enablers were grouped firstly into clusters or analytical themes. Afterwards, summary themes were iteratively developed by comparing findings across different study designs, contexts, and student populations. Any disagreements were resolved through discussion and consultation with another reviewer (CDN), when necessary. This process enabled the organization of findings for a more comprehensive understanding.

The narrative synthesis approach was particularly suited to the diverse set of qualitative, quantitative, and mixed-methods studies. This allowed for the integration of findings that would not have been suitable for meta-analysis due to methodological and contextual heterogeneity across the Sub-Saharan African region.

## Results

### Study selection

**Fig. 1:**
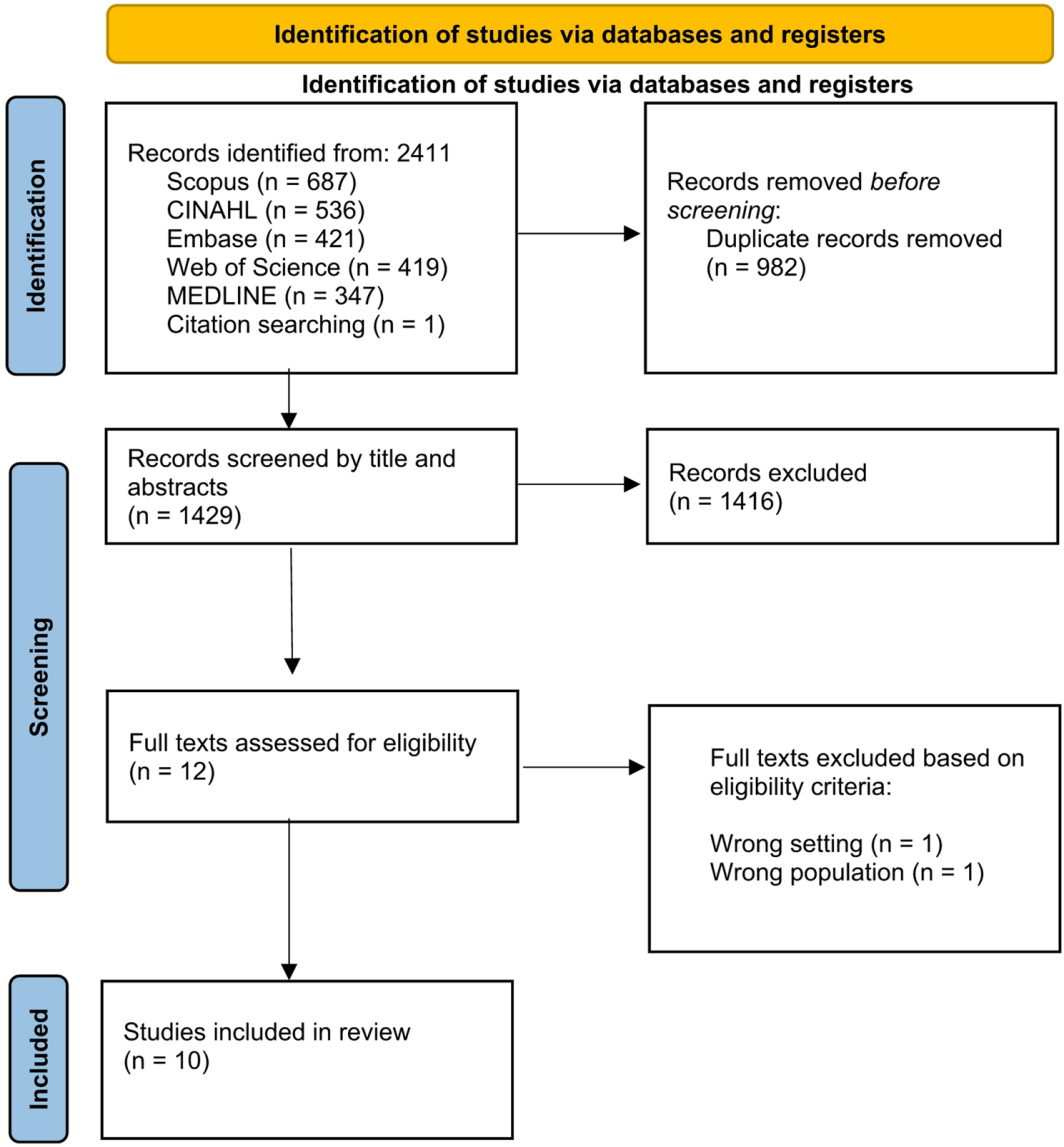
Study selection using the PRISMA flow diagram

Of 2411 studies identified from across the 5 databases, only 10 studies met the eligibility criteria. This study selection process is shown in Fig. 1.

### Study characteristics

The review included 10 studies across various Sub-Saharan African countries. Single site studies were conducted in Namibia (*n* = 1) [24], Uganda (*n* = 3) [6, 25, 26], South Africa(*n* = 2) [9, 27], Rwanda (*n* = 1) [28], Sudan (*n* = 1) [29], and Nigeria (*n* = 1) [18]. Additionally, one multi-site study was conducted across three universities in Uganda, South Africa, Sudan [17].

The studies consisted of various study designs, including 6 quantitative studies [6, 9, 18, 25, 28, 29], 3 qualitative studies [24, 26, 27], and 1 mixed-methods study [17]. Sample sizes varied considerably, ranging from 20 [24] to 1,815 participants [9]. Across all included studies, study participants represented various health professions located in different colleges, schools and departments across various universities. These included medicine, nursing/midwifery, pharmacy, dentistry, physiotherapy, occupational therapy, speech-language and hearing therapy, human nutrition, biomedical sciences, medical radiography, public health, and allied health professions (where unspecified in some cases).

These characteristics reflected the heterogeneous nature of research contexts and objectives across the studies. Thus, a meta-analysis was not conducted.

### Quality assessment

The quality of included studies was assessed using the Mixed Methods Appraisal Tool (MMAT) version 2018. Scores ranged from 60% (moderate quality) to 100% (very high quality) across the included studies. Of the 10 studies, five studies were rated as very high quality (100%) [9, 24, 27–29], three as high quality (80%) [6, 25, 26], and two as moderate quality (60%) [17, 18]. Notably, no studies were rated as low quality. This indicates a strong methodological quality of the included studies. Table 2 below shows a description of the characteristics of included studies and quality appraisal scores.

**Table 2:** Characteristics of the included studies and quality appraisal scores

S/*N*	AUTHOR, YEAR OF PUBLICATION	COUNTRY	STUDY AIM	STUDY DESIGN, SAMPLE SIZE	AGE RANGE, GENDER DISTRIBUTION	ACADEMIC YEAR, HEALTH DISCIPLINE	QUALITY
1.	Ashipala and Livingi 2021 [24]	Namibia	To explore and describe undergraduate nursing students’ challenges when writing research proposals at the University of Namibia (UNAM)	Qualitative, explorative, descriptive and contextual design. (semi-structured interviews) *N* = 20	18–30 years: 16 participants 31–40 years: 3 participants 41–50 years: 1 participant. 11 Males and 9 Female	Nursing 3rd year	*****
2.	Delport et al. 2023 [17]	South Africa, Sudan, Uganda.	To explore and compare the inclusion and impact of research training in undergraduate medical curricula across three African institutions; University of Pretoria, (South Africa); Al Neelain University (Sudan); Busitema University (Uganda).	South Africa: Cross-sectional study using mixed methods (focus group discussions, key informant interviews, and survey analysis). Sudan: Cross-sectional study using mixed methods (research project evaluations, faculty questionnaires, and curriculum document analysis). Uganda: Interventional study using quantitative methods (pre- and post-test questionnaires) Total: 41 faculty members and 554 students across all three studies. South Africa: 7 students in each FGD group, 3 KIIs, 275 (2015), 292 (2016), and 287 (2017) students in the survey. Sudan: 19 student research projects evaluated, 38 faculty members surveyed, and 167 students participated in module evaluations. Uganda: 72 students participated in the study.	Uganda: Mean age was 24 years, with a median age of 23 years and an interquartile range of 21–28 years. Nearly equal representation of male (50.4%) and female (49.6%) students. Other countries did not specify age ranges or specify gender distribution.	South Africa: Second to final year medical students. Sudan: Third to fourth year medical students. Uganda: Second-year medical and nursing students. Medical students across all three institutions; additionally, nursing students in Uganda.	***
3.	Kiyimba et al. 2022 [6]	Uganda	To assess research involvement of undergraduate students exploring awareness, barriers and motivators in all the 12 undergraduate HPS’ schools in Uganda. Makerere University (MAK), Mbarara University of Science and Technology (MUST), Busitema University (BU), Kabale University (KU), Gulu University (GU), Kampala International University (KIU), King Caesar University (KCU), Uganda Christian University (UCU), Muni University, Soroti University, Lira University, and Islamic University in Uganda (IUIU)	Cross-sectional Quantitative study(survey) *n* = 398	Mean age 23.9 ± 3.7 years 267 (67.1%) male, 131 (32.9%) female	Academic year: Year 1: 43 (10.8%) Year 2: 111 (27.9%) Year 3: 97 (24.4%) Year 4: 119 (29.9%) Year 5: 28 (7%) Health disciplines: MBChB: 220 (55.3%) BDS: 10 (2.5%) BNUR: 52 (13.1%) BPHARM: 44 (11.1%) Others: 72 (18.1%). Participants were from various health-related programs including Bachelor of Medicine and Surgery (MBChB), Bachelor of Biomedical Sciences (BSB), Bachelor of Nursing/Midwifery (BSN/MW), Bachelor of Pharmacy (BPHARM), Bachelor of Dental Surgery (BDS), Bachelor of Medical Radiography (BMR), and Bachelor of Science in Anesthesia (BSA).	****
4.	Awofeso et al. 2020 [18]	Nigeria	To examine the perceptions, attitudes, and the perceived barriers faced by medical students in Nigeria toward research at the College of Medicine, University of Lagos, Nigeria	Cross-sectional quantitative study *n* = 221 medical students (out of 835 distributed questionnaires, 26.5% response rate)	Age range = 17–34 years (mean 21.1 ± 2.8 years) Male: 128 (57.9%) Female: 93 (42.1%)	Preclinical (200–300 levels): 98 (44.4%) Clinical (400–600 levels): 123 (55.7%) Medicine (medical students)	***
5.	Marais et al. 2019 [27]	South Africa	To document the enablers and constraints of undergraduate research at Stellenbosch University Faculty of Medicine and Health Sciences (FMHS) and to explore how the presence or absence of choice influenced students’ engagement with research in this context.	Exploratory descriptive qualitative study using semi-structured interviews *n* = 21 participants total (10 students, 11 staff members)	All allied health student participants were female. MB, ChB staff participants were male; 1 of the four MB, ChB student participants was male. Human Nutrition, Occupational Therapy, Physiotherapy, Speech-Language and Hearing Therapy, and Medicine (MB, ChB) Females: 6 allied health students + 3 MB, ChB students = 9 females Males: 1 MB, ChB student + 8 MB, ChB staff members = 9 males So, the gender distribution among the 21 participants is 9 females and 12 males.	Students were primarily in their 4th year for allied health programs, while MB, ChB students typically conducted research in their 6th year.	*****
6.	Munabi et al. 2006 [25]	Uganda	To document the status of research among graduate and undergraduate students before the change to the new problem-based curriculum at Makerere University Faculty of Medicine.	Cross-sectional Quantitative Study (survey) *n* = 424 students (372 undergraduates, 52 postgraduates)	40% (169/424) female, 60% (258/424) male, 1% (7/424) did not indicate their sex	Medicine	****
7.	Mugabo et al. 2021 [28]	Rwanda	To describe the level of research involvement amongst undergraduate students at the College of Medicine and Health Sciences (CMHS) at University of Rwanda (UR) and to assess factors associated with research involvement.	Cross-sectional Quantitative Study (survey) *n* = 324	Age range: Mean age 23.3 years (standard deviation 2.27) Gender distribution: Males: 65.1% (*n* = 211) Females: 33.3% (*n* = 108). Preferred not to disclose: 1.5% (*n* = 5)	Academic year: Second year: 18.2% (*n* = 59) Third year: 47.2% (*n* = 153) Fourth year: 18.2% (*n* = 59) Fifth year: 6.2% (*n* = 20) Health disciplines: School of Medicine and Pharmacy: 46.6% (*n* = 151) School of Nursing and Midwifery: 28.1% (*n* = 91) School of Health Sciences: 10.2% (*n* = 33) School of Public Health: 9.6% (*n* = 31) School of Dentistry: 5.6% (*n* = 18)	*****
8.	Osman 2016 [29]	Sudan	To explore students’ perceptions, attitudes, motives and barriers toward research, and their perceptions on the quality of research supervision at University of Medical Sciences and Technology	Cross-sectional Quantitative Study (survey) *n* = 104	Age range: Mean age 22 ± 1.4 years Gender distribution: 45 (43.3%) males, 59 (56.7%) females	Final (5th) year medical students Medicine	*****
9.	Bovijn et al. 2017 [9]	South Africa	To quantify voluntary research involvement among medical and allied health professions (AHP) students at the Faculty of Medicine and Health Sciences, Stellenbosch University, South Africa, and to explore factors associated with such involvement. It also sought to determine if voluntary research involvement and/or other demographic factors are associated with self-perceived research competence and attitude toward future research participation.	Cross-sectional Quantitative Study (survey) *n* = 1815 (80.2% response rate)	Age Range: The median age for the whole group was 21 years, with a range of 17 to 37 years. Gender Distribution: Male: 463 (25.6%) Female: 1339 (73.8%) Not specified: 13 (0.6%)	undergraduate programme in Medicine and Surgery (MB, ChB – a 6 year programme); as well as four undergraduate AHP programmes: Bachelor of Science (BSc) in Dietetics (DT), Bachelor of Occupational therapy (OT), BSc in Physiotherapy (PT), and Bachelor of Speech, language and hearing therapy (SLHT), all 4 year programmes.	*****
10.	Wakida et al. 2022 [26]	Uganda	To evaluate the perceptions of undergraduates and mentors on the appropriateness, acceptability, and feasibility of the HEPI-TUITAH Micro-Research Approach to HIV Training in Uganda: Mbarara University of Science and Technology (MUST), Bishop Stuart University (BSU), and Lira University (LU).	Cross-sectional descriptive qualitative study *n* = 24 undergraduate health students and 13 mentors in 5 focus group discussions (5–8 participants per group)	Not specified	Not specified	****

5***** or 100% quality criteria met

4 **** or 80% quality criteria met

3 *** or 60% quality criteria met

2 ** or 40% quality criteria met

1 * or 20% quality criteria met

### Research activities

To systematically categorize the diverse research activities identified across the included studies, we adapted an already established research process framework [23]. This adaptation involved consolidating their original phases into three distinct phases to better reflect the undergraduate research experience:

- Early Phase: Encompasses the original conceptual, design, and planning phases, focusing on foundational research preparation, proposal development, grant writing and obtaining ethical approval.
- Middle Phase: Combines the empirical and analytical phases, covering active data collection, analysis and management activities.
- Late Phase: Corresponds to the dissemination phase, involving scholarly communication and knowledge translation activities including writing of manuscripts, publication of peer-reviewed articles, writing abstracts and presenting at conferences.

This classification facilitates a comprehensive understanding of how undergraduate health students engage in research activities. See Table 3 below.

**Table 3:** Research activities undertaken by UHS in SSA

Phases of research	Research activities mentioned	No of studies	References
Early Phase	Research proposal writing	6	[6, 17, 24, 26, 28, 29]
	Study design and protocol development	5	[17, 26–29]
	Literature review	4	[17, 24, 26, 27]
	Grant writing	2	[26, 28]
	Ethics approval	5	[24, 26–29]
Middle Phase	Data Collection	5	[6, 17, 25, 26, 28]
	Data Analysis and Management	5	[9, 17, 26, 28]
Later Phase	Manuscript Writing	5	[6, 26–29]
	Publication of peer-reviewed articles	7	[6, 9, 17, 18], 26– [29]
	Writing abstracts and presenting at conferences	5	[6, 9, 18, 26, 28, 29]

#### Other research activities

The studies also document the various roles that UHS play in participating in research activities. Four studies document research assistant roles as a common entry point to research [6, 9, 25, 28]. In one study, the findings report that 61% of UHS who participated in that study had served as research assistants at one point in their research journey [25]. Other roles include first author positions in a published paper [6, 26, 28], co-authors [6, 25, 28, 29] and as principal investigators [6, 25].

Interestingly, UHS were also involved in curricular research and voluntary research depending on the contexts. In the multisite study by Delport et al. [17], findings reveal different approaches to curricular research integration in three contexts (South Africa, Sudan, and Uganda). In Uganda, UHS were involved in a one-week research methods training module, followed by an application of research skills in a community setting thereafter. In contrast, UHS in South Africa participated in research modules during their second year, which entailed literature reviews, protocol development, and research report writing. While in Sudan, students in the third year participated in research methodology courses followed by individual research projects in the fourth year. On the other hand, in a Nigerian study, about 52.9% of participants reported participating in a voluntary research project [18]. This finding suggest that research participation could extend the traditional “mandatory research projects” style offered in institutions.

### Barriers to research participation

The barriers to research participation among UHS were grouped into 7 themes: Resource Constraints; Time and Curriculum pressures; Knowledge and skills gap; Administrative, Ethical and Operational Hurdles; Mentorship and Supervision Challenges, Perceptions and Attitudes to Research; Gender, Language and Cultural barriers.

#### Resource constraints

Across the included studies, financial challenges were identified as the most common barrier reported by UHS in six studies [6, 9, 17, 24, 27–29]. Beyond financial limitations, UHS also reported inadequate infrastructure including limited library resources and the lack of well-equipped laboratory [24] and computer facilities [18, 29]. In another study, students reported a poor awareness of available research resources [27].

#### Time and curriculum pressures

Time related constraints constituted the second most prominent barrier theme. Seven studies reported difficulty in balancing personal life, academic work, and research, coupled with insufficient time allocation for research [6, 17, 18, 24, 25, 27, 29]. The demanding nature of curricula for health professions education was another common barrier, coupled with overwhelming workloads [18, 24, 26, 27, 29]. With these constraints, UHS were not likely to have ample dedicated time for research.

#### Mentorship and supervision challenges

In 6 studies, UHS expressed a lack of professional supervisors and proper mentorship/guidance needed to support their research process [6, 17, 24, 25, 28, 29]. In some studies, UHS described experiences of poor communication with research supervisors [17, 24, 26], and in others, they felt the supervisors were not fully committed and unwilling to supervise [17, 27, 29]. In the backdrop of such challenges, supportive specialized staff such as biostatisticians, bioethicists and editors were lacking [29].

#### Knowledge and skills gap

For this theme, UHS reported a reported lack of knowledge about the research process [24, 28, 29], in three studies. In addition, two studies identified inadequate research and biostatistics curriculum [18, 29]. On the skills gap, UHS in some studies reported an absence of structured content on research [27], a lack of mandatory courses on research methodology [6], lack of statistical support [9], and poor research skills [17], as key barriers.

#### Administrative, ethical and operational hurdles

Even upon receiving the skills they needed for research, UHS also faced another hurdle: difficulty gaining administrative and ethical approval for their research. UHS faced lengthy approval processes and burdensome requirements [9, 24, 27, 29]. Likewise, they reported the research activities as not being well organized [17]. Asides these challenges, UHS reported some operational challenges that may exist outside of their control. In one study, COVID-19 pandemic restrictions deterred research participation among UHS [26]. Other times, UHS faced difficulties in selection of research topics, patient follow-up, and availability of sample/study subjects [17, 24, 29]. Other barriers identified include the lack of collaborations and recognition of research efforts [6, 18, 25].

#### Perceptions and attitudes to research

UHS had varying perceptions and attitudes towards research, that constituted this barrier theme. In three studies, UHS described research as complex, difficult and stressful [6, 17, 18]. In some studies, participants reported a lack of interest and motivation for research [6, 18, 29]. In one study, research anxiety and fear of conducting independent research was reported as barriers to research participation [17]. Another concerning finding was that UHS felt unqualified to do research [28].

#### Gender, language and cultural barriers

The most pronounced finding within this theme relates to significant gender disparities that exist in research participation. Considering one of the included studies with the largest sample size (*n* = 1,815), they found that male UHS were significantly more likely to be involved in voluntary research compared to female UHS, with an odds ratio of 1.99 (95% CI: 1.48–2.67; *p* < 0.001) [9]. This finding was corroborated by Mugabo et al. [28], who reported that more male UHS (52.6%) had participated in research projects compared to female UHS (43.5%, *p* = 0.023). However, in the study by Bovijn et al. [9], they found that female UHS reported lower self-perceived research competence and demonstrated reduced interest in future research participation when compared to their male counterparts. Language and cultural barriers emerged as significant challenges that may affect research participation barriers within research teams [9, 25, 26]. Specifically, Wakida et al. [26] identified communication challenges due to language barriers within research teams as a key impediment to effective collaboration and research participation.

### Enablers/facilitators of research participation

We found various enablers or facilitators of research participation among UHS in SSA reported in the included studies. These were grouped into 6 themes; Early Exposure to research and Integration in curriculum; Quality mentorship and supervision; Access to Funding and Resources; Research skill development; Individual Motivation and Professional Identity; and Support from groups and institutions.

#### Early exposure to research and integration in curriculum

This was cited as the most common facilitator theme across the included studies. Six studies cited the inclusion of research in the early years of undergraduate study as prominent facilitators of research participation among UHS [6, 17, 18, 24, 27, 29].

The value of structured curriculum approaches was emphasized by findings supporting structured time allocation for research [25, 27], mandatory research components [29], and problem-based learning curricula [18]. Likewise, completion of curricular research projects and hands-on research experience were also identified as enabling factors [25, 26]. Additionally, participants who had previous research experience were likely to participate in future research projects [9, 18, 25].

#### Quality mentorship and supervision

Within this theme, UHS emphasized the importance of a role model/mentor to provide guidance [6, 17, 25, 26, 28]. Nevertheless, two studies [6, 24] highlighted that proper guidance extended to support with topic selection, manuscript writing, and publication processes, with faculty mentoring UHS as well [28].

Similarly in five studies, UHS highlighted the need for supportive supervisors [6, 24, 27–29]. In a particular study, the aspects of quality supervision were emphasized, as supportive supervisors possessing knowledge, experience, and research skills to assist in research facilitated the engagement of UHS in research [29].

#### Access to funding and resources

While resource and funding constraints acted as barriers, studies reported that access to adequate funding and resources was also an important facilitator. Enablers reported here included a call for funding for research projects [6, 25, 26, 28]. Improved internet access and data availability [24, 25], as well as the provision of library resources [25] were also recognized as enabling factors.

#### Research skills development

Three studies emphasized the value of research methodology training, including short courses on research methodology, data analysis, proposal development, and manuscript writing [6, 17, 26]. Similarly, library information literacy [24], practical application of research during community placements [17], hands-on training [26], and pre-dissemination training [26] were seen as crucial facilitators.

#### Individual motivation and professional identity

This theme encompasses both the internal psychological drivers and professional aspirations that motivate UHS to engage in research.

Recognition and rewards emerged as powerful motivators that validate UHS’ research efforts and provide tangible benefits for participation. Acknowledgement of research achievements was reported as an enabler by two studies [6, 27], and monetary rewards was identified by another study [6]. However, benefits exceed monetary benefits and could include opportunities to attend or present at a conference [26, 28], as well as publication opportunities [26].

Participants’ perceptions of benefits of research participation could serve as their motivation. In two studies, UHS perceived that research experience may increase their acceptance into residency programs [6, 29]. In other studies, UHS perceived research as important and relevant to personal development, clinical work and careers [6, 25, 26, 29]. Additionally, the positive attitudes towards research and willingness to learn were identified as a fundamental motivator to research participation [18]. These findings may suggest that students with positive research attitudes and perceptions approach research challenges with resilience and view difficulties as learning opportunities rather than insurmountable barriers.

#### Support from groups and institutions

As reported by Wakida et al. [26], the interprofessional education approach (the approach of bringing together students from different health disciplines) was seen as valuable for peer mentorship, teamwork, and academic collaboration. Other studies reported collaboration with other researchers [6], involvement in ongoing research projects [28], working in groups [27], and peer collaboration [28] as enablers to research participation. These findings suggest that research participation could be enhanced when students perceive it as a collaborative rather than isolated activity.

Maintaining a suitable institutional research environment was identified as essential to supporting research participation and growth [6, 27, 28]. In essence, these could include the establishment of undergraduate research support centers [28] and facilitating the complex hurdle of ethics and institutional permissions processes [27]. With the creation of such support centres and streamlining ethical approval processes, UHS can face lesser barriers to participating in research activities.

## Discussion

This systematic review synthesized evidence on the barriers and enablers to research participation among UHS in SSA. The findings reveal that UHS’ research participation is predominantly shaped by a myriad of structural and institutional conditions (funding/resources, mentorship and supervision, administrative/ethical approval processes, curriculum and time), with individual level factors playing a somewhat secondary, but also crucial role. In this review, participation in research activities were grouped according to phases ranging from early, middle and later phases.

Findings were synthesised focusing on the most frequently reported institutional and structural factors, as these appear most influential across contexts. Interestingly, some factors were reported as both barriers and enablers depending on the context. Thus, these findings carry important implications for policymakers, researchers, practitioners, and health professions education systems at large.

### Barriers to research participation

####  Resource constraints

Majority of the included studies describe resource constraints as a common barrier to research participation among UHS. For example, UHS report that limited access to research funding and research infrastructure including laboratories and libraries as resource constraints. This finding suggests that without adequate resources, material, and otherwise financial, UHS, who represent future health researchers are unable to take action that translates from mere research interest to practical engagement in research activities. These results further indicate that structural and system-level investment in research support is crucial for research participation. Our findings share similar patterns, consistent with another study on research participation among medical students, which identified the lack of research funding as critical barriers to research participation among students in India and Saudia Arabia respectively [30, 31].

####  Time and curriculum pressures

Across the studies, UHS report difficulty in balancing the demanding nature of their clinical rotations and coursework with research activities. These findings closely align with previous research published in 2020 and 2024, which highlighted that UHS often struggle to conduct research while managing the academic pressures of their education, leading to reduced completion and participation rates in research [32, 33]. These tight curriculums, in addition to exams, heavy course loads, and the lack of protected time for engagement in research suggests that UHS may struggle to start and more importantly, sustain their involvement in research projects. In contrast, findings from a study conducted among medical students in India revealed 55.79% of respondents agreed that engaging in research was not a waste of time and did not interfere with their academic studies [30]. This contrasting finding indicates that in cases where UHS see value in engaging in research, they may be often more willing to invest their ‘limited time’. In all, these findings support the call for institutions to embed opportunities to participate in research such as research modules with designated time periods to allow students participate actively without threatening other academic responsibilities.

####  Mentorship and supervision challenges

Challenges regarding mentorship and supervision were reported as additional barriers in several studies. In particular, UHS described poor communications with supervisors and perceived a lack of commitment from assigned mentors to the research projects. This finding points to well-known gaps in institutional capacity, where there may be either few faculty assigned to large number of students or absence of mentorship programs to offer structured guidance to students on research [34, 35]. This unavailability of these supportive structures may leave UHS without proper guidance on how to choose right research design or methods needed for their research. These results are consistent with a cross-sectional study and a systematic review which identified limited access to mentorship, supervision and guidance as key barriers to UHS involvement in research [35, 36].

####  Administrative, ethical and operational hurdles

UHS encountered significant challenges in executing their research projects. One of such hurdles was the difficulty in obtaining ethical and administrative approvals for research topics and proposals. These processes were often characterised by prolonged processing times, extensive requirements and burdensome procedures. Consistent with another study with medical students, difficulties obtaining ethical or institutional approval posed a significant barrier to engagement in research [36]. This implies that existing institutional systems intended to protect research participants, may act as gatekeepers deterring research participation among UHS, as delays in obtaining ethical and institutional approval can further result in delays in data collection.

####  Gender, language, and cultural barriers

Although less frequently reported, our findings also highlight how certain sociocultural factors remained significant barriers hindering research participation. Across the diverse sub-Saharan African contexts, gender disparity was a notable barrier, in that male UHS were more likely to engage in research activities than their female counterparts. The underlying factors resulting in these gender disparities, ought to be viewed as structural factors that extend beyond individual attitudes or interest in research. However, gender disparities are context specific. In contrast to our findings, a South Arabian study found that female medical students, had higher participation rates, when compared to their male counterparts. This was attributed to targeted efforts to support female students in that context [37]. This suggests that female students participate equally (or more) in research, when provided with the right opportunities and encouragement. In addition to gender-based differences, language and cultural barriers were identified as significant impediments to effective research participation. Communication challenges within research teams, particularly those stemming from language differences, may hinder collaboration and limit opportunities for meaningful engagement in research activities.

### Facilitators to research participation

#### Early exposure to research and integration in curriculum

In a number of studies, early research exposure was reported as a facilitator, often in the form of embedded research activities, required projects, or structured opportunities within training programmes. For instance, some studies found that UHS who had early exposure with research were more likely to participate in future research activities. This finding suggests that early exposure familiarizes UHS with research processes and reduces unease often associated with starting research tasks. The results further revealed that exposure to research early-on, can later shape or expose UHS to career trajectories in research and academia. Similarly, findings from Indonesian study revealed that about 76.35% dental students felt that their early exposure to research motivated them to pursue their academic careers and also provided transferrable skills, which could be seen as tangible benefits for participation [38].

#### Quality mentorship and supervision

Findings from our review also revealed that UHS students consistently emphasised the vital role of having a mentor or role model to provide structured and sustained guidance throughout the research process. UHS repeatedly highlighted the value of supportive supervision, particularly when supervisors demonstrated not only encouragement but also strong research expertise, experience, and academic competence. Notably, a mixed-method study affirmed the transformative impact of such mentorship on undergraduate research experiences. Given the mentorship, students reported increased confidence and a strengthened belief in their ability to undertake and contribute meaningfully to research tasks [39]. This reflects the role of mentorship in providing research guidance and motivational support, calling for institutional investment in training for mentors and supervisors. In the same light, this calls for the good practice of pairing UHS with well trained quality mentors towards significantly enhancing UHS research participation.

#### Access to funding and resources

While resource and funding constraints were identified as significant barriers, several studies also highlighted the availability of adequate funding and resources as key facilitators of research engagement. Additional facilitating factors within this theme included improved internet connectivity, enhanced data accessibility, and the provision of adequate library and learning resources. These findings are consistent with another study, which affirm that the availability of appropriate research tools and software served as a strong motivator for both students and faculty members to participate in research activities and pursue deeper academic inquiry [40]. This portrays that the availability of resources provides tangible means to support research processes from data collection to dissemination, reducing persistent logistical barriers.

#### Institutional support

A conducive institutional research environment was highlighted as crucial for fostering active participation and the growth of research capacity among UHS. Key strategies include the establishment of undergraduate research support centres and the simplification of processes related to ethical approvals and institutional permissions. These measures can significantly reduce administrative barriers and enhance students’ ability to participate in meaningful research activities. Consistent patterns are observed in another review, emphasizing the importance of institutional support, highlighting that structured support and good research culture embedded in institutions was a significant enabler for medical students’ research involvement [33]. This finding suggests that institutional expectations and cultures that value research can normalise research participation, making it a standard part of professional practice rather than an optional extra.

Together, these facilitators and barriers suggest that interest and individual motivation alone is not sufficient. Thus, cultivating and investing in system level supports, enabling environments, and formalized research structures are essential to foster research participation across sub–Saharan Africa.

### Patterns of research participation

The findings demonstrate an uneven distribution of UHS’ engagement across the research phases, with participation often concentrated in early phase activities such as (research topic selection, proposal writing) and notably declining in the middle phases (data collection and analysis), and later phases of manuscript writing. Interestingly, UHS were also involved in co-authoring publications and other dissemination activities. These early phase activities appear to serve as the most accessible entry points into research, largely because they are commonly embedded within undergraduate curricula or mandatory final-year projects. Conversely, participation notably declines in the middle phase, suggesting that with activities like data collection, analysis, and management, there is a need for more time commitment, resource availability (funding, software, laboratory among others). This will in turn increase competences in research methodology. This pattern of inconsistent engagement across the research phases suggests that without structural support towards research involvement, institutions may fail succeed to sustain research participation, even after introducing students to the idea of research.

The observed pattern of uneven engagement across research phases is supported by other studies which document challenges and successes in research participation among UHS. Studies across diverse settings have found that the lack of time, inadequate mentorship, insufficient funding coupled with limited research knowledge and skills, constitute critical barriers that prevent students from sustaining research involvement beyond the early phases [32–36]. Consistent with our findings, a study from Canada, demonstrated that scaffolded, multi-year course-based undergraduate research training implemented within a well-resourced institution supported with sustained mentorship, and structured skill-building found success in maintaining student engagement across several research phases [41].

### Strengths and limitations

The inclusion of studies from multiple countries and health disciplines offers a rich and well-structured view of undergraduate research activities. This review is grounded in strong methodological principles, following PRISMA 2020 guidelines, PROSPERO registration, comprehensive database searches, and systematic quality appraisal using the MMAT. By providing insights from UHS perspectives, this review offers practical, context-sensitive recommendations that speak directly to the realities of UHS research participation in Sub-Saharan Africa.

However, the uneven geographical coverage limits the generalisability of the findings to the entire SSA region, and restricting the search to English-language publications may have introduced language bias by excluding potentially relevant studies in French, Portuguese, or other local languages. The heterogeneity in study designs, participant populations, and contexts necessitated a narrative rather than quantitative synthesis, the former of which is more susceptible to interpretive bias. The exclusion of grey literature and reliance solely on peer-reviewed sources may have also overlooked important but unpublished perspectives, particularly from low-resource settings. In addition, many of the included studies relied on self-reported data, which are inherently vulnerable to recall and social desirability biases. It is also acknowledged that restricting the search to only five databases may have resulted in the omission of relevant studies not indexed in these databases. Finally, the temporal scope (2000–2024) may have excluded older but still relevant evidence from countries with long-established traditions in undergraduate research.

### Implications for research, practice and policy

This systematic review finds that sometimes, barriers and enablers can be two sides of the same coin, in regard to research participation among UHS. Therefore, this section outlines critical areas requiring the attention of policymakers, practitioners and researchers to enhance UHS participation in research across Sub-Saharan Africa.

This review found that majority of the studies were of a quantitative design, with only three qualitative studies and one mixed methods study. The quantitative studies reported high levels of interest in participating in research activities, however, actual participation rates remain low. This divergence suggests that complex mediating factors exist in the pathway from interest/intention to action, which may not be adequately captured when using only quantitative methods. Thus, we encourage researchers to carry out more mixed methods and qualitative studies to explore the deeper contextual factors and underlying mechanisms that influence UHS’ research participation. Qualitative research particularly can shed more light on how students navigate structural and institutional barriers, decision making about research involvement and competing academic workloads across diverse contexts. Such would benefit from long term research in form of longitudinal study designs that follow up with students from their earlier undergraduate years through to final year and graduation. This will capture how participation patterns evolve over time. Furthermore, the review found only one study that compared research participation across 3 SSA countries. Researchers are thus encouraged to consider collaborations with other SSA institutions to conduct the much-needed comparative research, in order to examine how factors affecting research participation vary across different educational and resource contexts in SSA. This would improve current understanding of national and institutional contextual factors that shape research participation trajectories across SSA, with lessons learnt to be applied in other contexts globally. Again, participatory research with UHS as co-researchers is encouraged to challenge traditional research hierarches where research is done “on” or “for” the people rather than “with” them, creating avenues for equitable decision-making, and unique perspectives. This will enable the co-production of research that is meaningful, grounded in real life contexts, and relevant to policy and practice.

From a practice perspective, health professions educational institutions should carefully consider the barriers and facilitators identified in this review, within their specific institutions, in order to implement evidence-informed interventions to promote research participation among UHS. This review identified different solutions that practitioners, including educators, research offices, and academic programme leads can work together to implement. These promising solutions include the provision of structured mentorship programs, supportive research staff and integrating curriculum-based research training within their institutions. As the findings suggest, these solutions that offer early exposure to research can result in an increased participation in research activities, development of skills, and spur UHS for a career in research and the academia. Likewise, practitioners must target bottlenecks within administrative, ethical and/or institutional approval processes that disproportionately disadvantage UHS researchers within their institutions. They can consider the implementation of student friendly ethics pathways that provide adequate support while maintaining rigorous standards. This includes gender-sensitive and supportive supervision, developing templated ethics application documents, implementing predictable review cycles with specified turnaround times, providing ‘proportionate’ review for minimal risk studies to avoid overburdening students with complex requirements, and conducting ethics training sessions frequently, which would be tailored to student needs.

Current approaches often emphasize student motivation and individual intent to participate as drivers of research participation. However, evidence from our review suggest that while these play a role, structural and institutional factors may play a more crucial role in creating the conditions where students are able to get involved (or not) in research activities. Therefore, policy responses must prioritize creating enabling institutional conditions and research infrastructure that cultivates a supportive research culture for promoting active engagement in research among UHS. Policymakers at national and regional levels should establish and institutionalize dedicated research funding streams for students and early career researchers. These funding streams could be supported through national research councils, which will ensure sustainability and mandate transparency of resources. Furthermore, educational policies should explicitly prohibit practices that are exploitative, such as excluding students’ name in research publications, when those students have made substantial contributions to the research. Also, the practice of supervisors requiring students to include the names of other colleagues who have not meaningfully contributed to the research for publication, should be curbed by policy. Such measures will protect student researchers and promote transparency and fairness in research and academic knowledge production.

## Conclusion

This systematic review highlights the complex interplay of barriers and enablers influencing research participation among UHS in Sub-Saharan Africa. While constraints such as limited funding, inadequate mentorship, insufficient research skills, curriculum overload, and bureaucratic challenges hinder participation, several facilitators such as early curriculum integration, quality supervision, access to research training, collaboration opportunities, and institutional support emerge as critical enablers. Notably, some factors like mentorship and resource availability function as either barriers or enablers depending on the context, underscoring the need for tailored interventions. To strengthen undergraduate research engagement in SSA, we recommend embedding research early in curricula, enhancing mentorship structures, improving access to resources and funding, simplifying ethics and administrative processes, fostering interdisciplinary collaborations, offering targeted research training, and addressing gender and cultural disparities. These efforts are essential to building sustainable research capacity and developing a future health workforce equipped to respond to local and global health challenges.

## Data Availability

All data analysed in this study were extracted from previously published studies, which are cited in the manuscript. No new datasets were generated. Therefore, data sharing is not applicable.
